# Supplementary material for: Drug screen reveals new potent host-targeted antivirals against Mpox virus
Source: bioRxiv. 2025 May 5:2025.05.02.651913. Preprint. [Version 1] doi: 10.1101/2025.05.02.651913 (PMC12094526; doi:10.1101/2025.05.02.651913)
Supplement: Supplement 1 [file media-1.pdf]

SUPPLEMENTAL FILE

Drug screen reveals new potent host-targeted antivirals against Mpox virus

Arjit Vijey Jeyachandran, Anne K. Zaiss, Nikhil Chakravarty, Sneha Singh, Yennifer Delgado, Ramya Paravastu, Nivedha Satheeshkumar, Ephrem Gerald, Aakash Jeysankar, Joshua Thomas, Lilly Fuller, Noella Lee, Cameron Taylor, Shantanu Joshi, Mark Parcells, Samuel W. French, Abhijit Date, Mehdi Bouhaddou, Gustavo Garcia Jr, Ashok Kumar, Robert Damoiseaux, Vaithilingaraja Arumugaswami

SUPPLEMENTAL FIGURES AND LEGENDS

Supplementary Figure 1

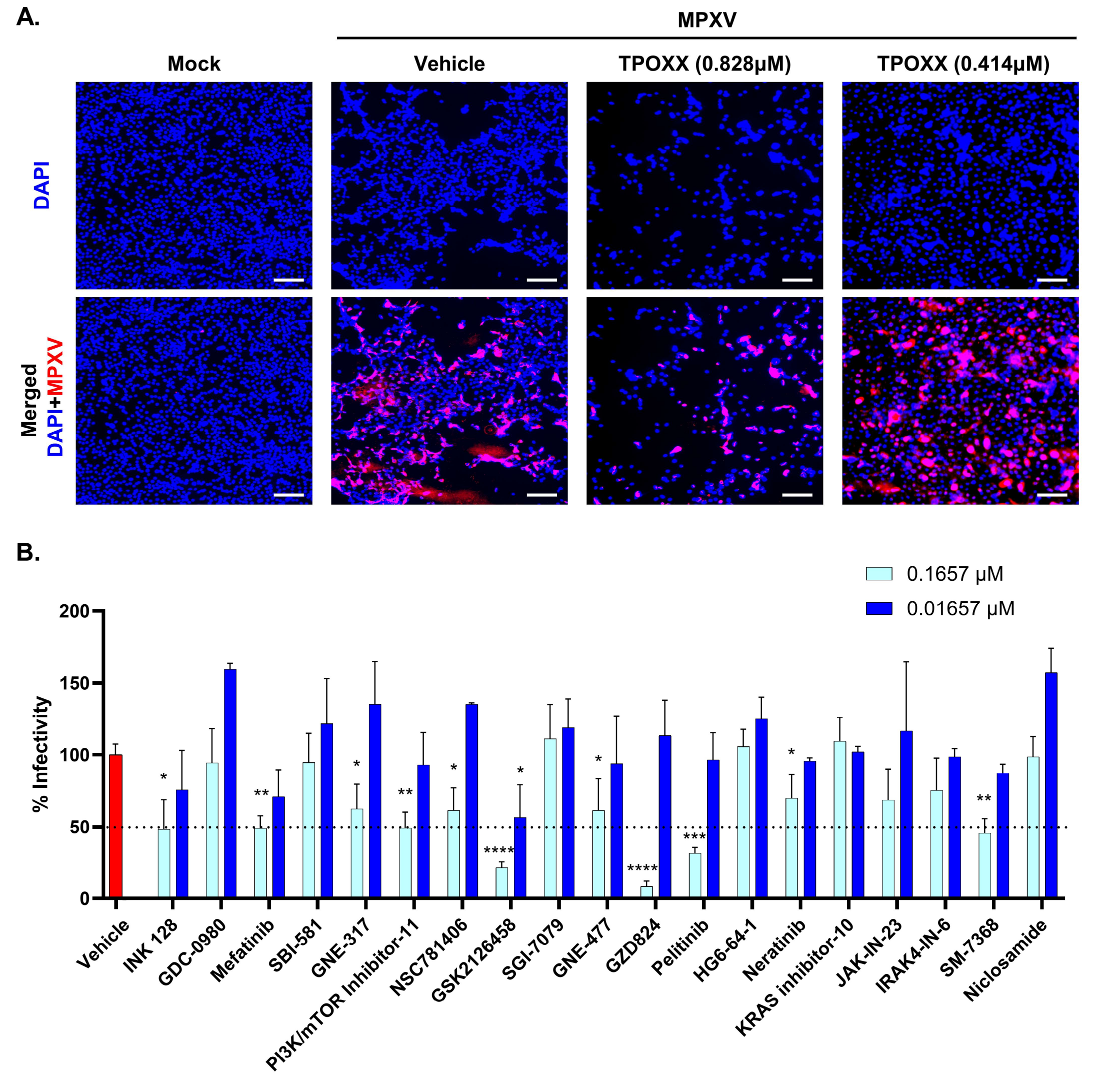

Supplementary Figure 1. A) *Immunofluorescence images of MPXV-infected retinal pigment epithelial cells, ARPE-19 (red) treated with Tecovirimat (TPOXX) at 0.828 μM and 0.414 μM for 48 hours post-infection (hpi). DAPI (blue) was used to counterstain nuclei. Scale bar = 100 μm.* ***B)*** *Quantification of MPXV-infected cells based on immunofluorescence imaging following treatment with 19 selected compounds from the secondary screening at 0.1657 μM (light blue) and 0.01657 μM (dark blue) for 48 hours post-infection (hpi). Percent infectivity was calculated as the ratio of MPXV-positive cells (red) to total DAPI-stained nuclei (blue) and normalized to the average infectivity of the vehicle-treated control group. The dashed horizontal line indicates the 50% infectivity threshold. Data represent the mean ± standard deviation (SD) from image-based quantification across three wells per condition. Statistical analysis was performed using one-way ANOVA, followed by Tukey's post hoc test (*P < 0.05; **P < 0.01; ***P < 0.001; ***P < 0.0001).*

Supplementary Figure 2

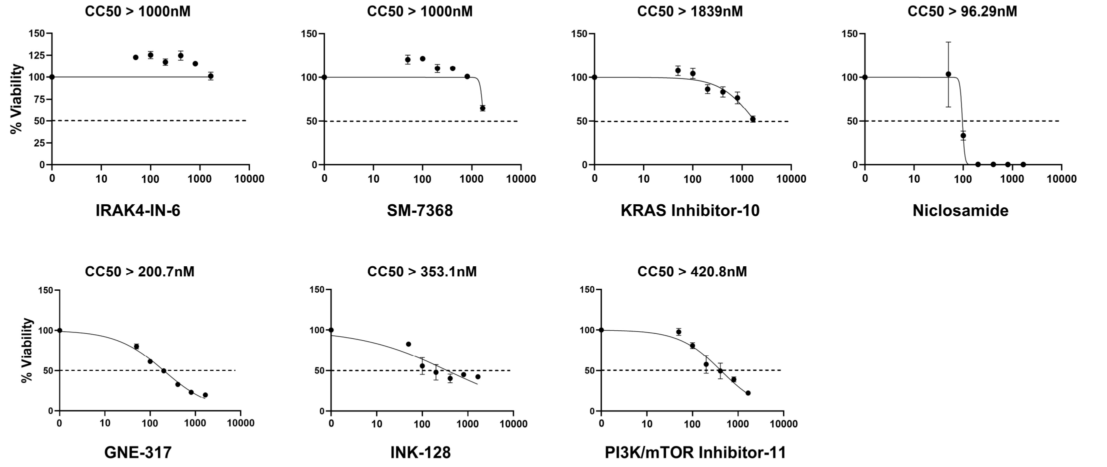

Supplementary Figure 2. *Cell viability of VK2/E6E7 human vaginal epithelial cells treated with seven selected drug compounds was assessed using the CellTiter-Glo Luminescent Cell Viability Assay. Cells were incubated with each compound for 48 hours, and luminescence was measured to quantify intracellular ATP levels, which correlate with metabolically active (viable) cells. Dose–response curves were generated to determine the half-maximal cytotoxic concentration (CC_{50}) for each compound. IRAK4-IN-6, SM-7368, and KRAS inhibitor-10 exhibited low cytotoxicity with CC_{50} values >1000 nM, while Niclosamide demonstrated higher cytotoxicity (CC_{50} = 96.29 nM). GNE-317, INK-128, and PI3K/mTOR Inhibitor-11 showed moderate cytotoxic effects with CC_{50} values of 200.7 nM, 353.1 nM, and 420.8 nM, respectively. The dotted horizontal line indicates the 50% viability threshold, and error bars represent the standard deviation (SD).*

Supplementary Figure 3

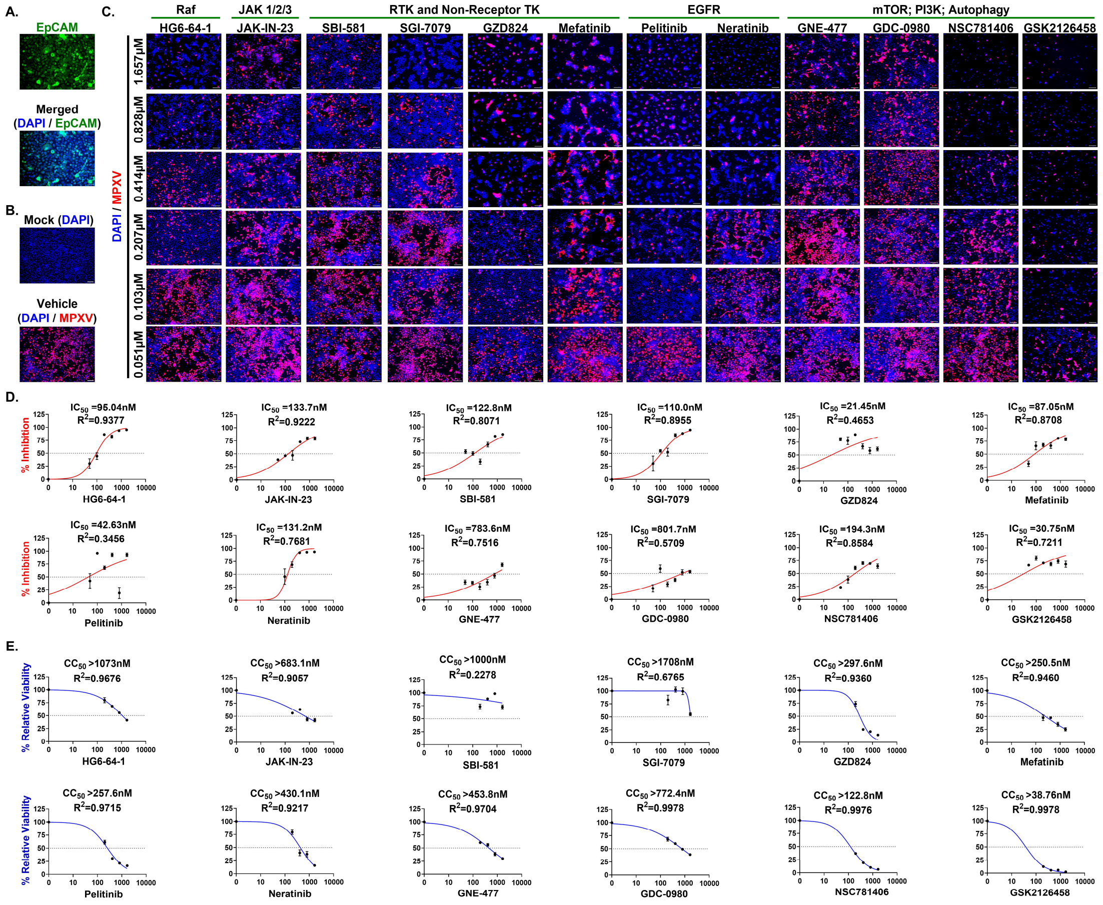

Supplementary Figure 3. A) *Immunofluorescence staining of the epithelial cell marker EpCAM (green) in human vaginal epithelial (VK2/E6E7) cells. Nuclei were counterstained with DAPI (blue). The merged image confirms epithelial cell identity. Scale bar = 100 µm.* ***(B)*** *Control conditions: Mock (uninfected) and Vehicle (MPXV-infected, untreated) wells stained for MPXV A4L viral antigen (red) and DAPI (blue) to visualize nuclei. These controls serve as reference for infection and treatment comparison. Scale bar = 100 µm.* ***(C)*** *Immunofluorescence images of MPXV-infected VK2/E6E7 cells following treatment with 12 drug compounds at six dose concentrations (1.657, 0.828, 0.414, 0.207, 0.103, and 0.051 µM). Cells were fixed at 48 hours post-infection (hpi) and stained for MPXV A4L antigen (red) and nuclei (DAPI, blue). The compounds are grouped by host target classes: Raf, JAK1/2/3, RTK and Non-Receptor Tyrosine Kinases, EGFR, and mTOR/PI3K/Autophagy, as labeled above each set. Scale bar = 100 µm.* ***(D)*** *Dose–response curves showing percent inhibition of MPXV infection in VK2/E6E7 cells treated with 12 compounds across six concentrations. Cells were infected with MPXV and fixed at 48 hours post-infection (hpi) for immunofluorescence analysis. Infection levels were calculated as the ratio of MPXV-positive (red) to DAPI-stained (blue) nuclei, normalized to vehicle controls.* IC_{50} *values were determined using nonlinear regression. The dotted line indicates 50% inhibition.* ***(E)*** *Dose–response curves showing relative cell viability for the drug treatments. Viability was assessed based on DAPI-positive nuclei counts, normalized to mock-treated (uninfected) controls.* CC_{50} *values were calculated using the top four concentrations for curve fitting. Data are presented as mean ± SD from triplicate wells. The dotted line indicates 50% viability.*

Supplementary Figure 4

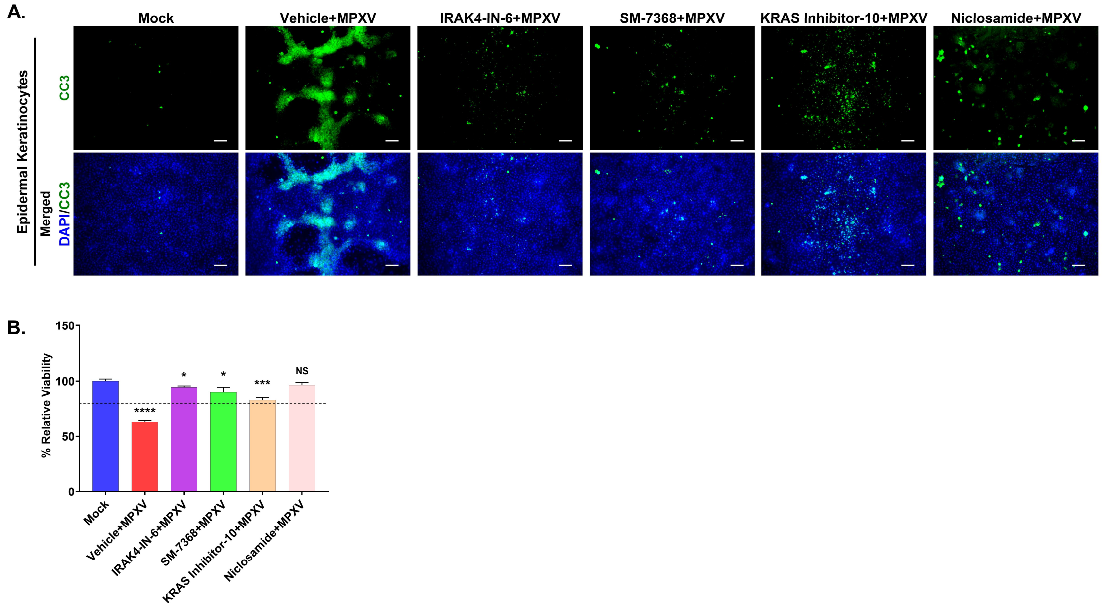

Supplementary Figure 4. A) *Immunofluorescence staining of human epidermal keratinocytes pre-treated with four selected drugs (IRAK4-IN-6, SM-7368, KRAS inhibitor-10, and Niclosamide) at 1.65µM, followed by MPXV infection and incubation for 48 hours post-infection (hpi). Cleaved caspase-3 (CC3, green) was used as a marker for apoptotic cells. Scale bar=100 µm.* ***(B)*** *Quantification of relative cell viability in MPXV-infected keratinocytes treated with four selected drug compounds. Viability was assessed by counting DAPI-stained nuclei in each condition and normalizing to the average DAPI cell count of mock (uninfected, untreated) wells. Data are presented as mean ± standard deviation. Statistical analysis was performed using ANOVA, followed by Tukey's post hoc test (*P < 0.05; **P < 0.01; ***P < 0.001; ****P < 0.0001; NS, not significant).*

Supplementary Figure 5

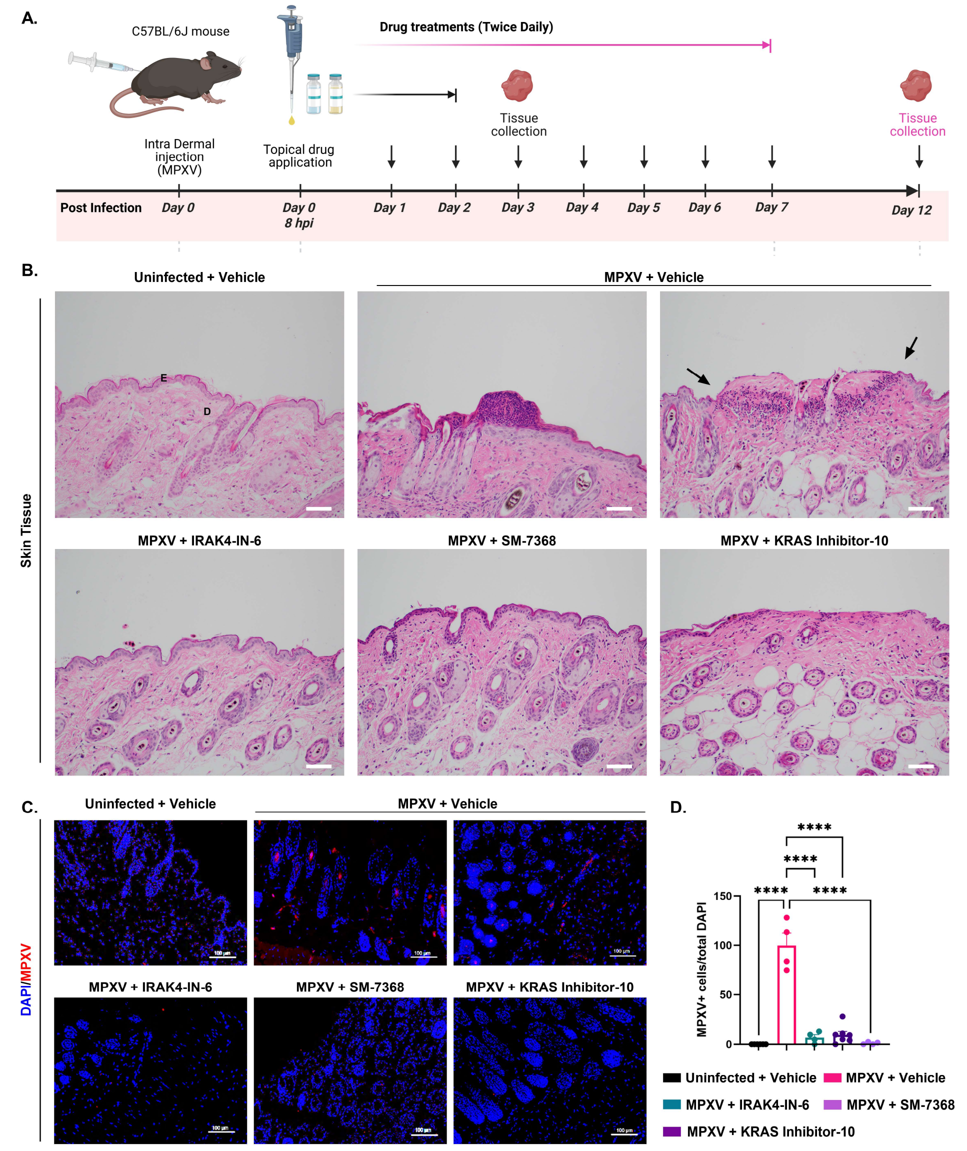

Supplementary Figure 5. A) *Schematic representation of the experimental workflow, illustrating the MPXV infection timeline, drug administration period, and tissue collection timepoints with day 12 highlighted in pink to indicate the focus of this supplementary figure.* ***(B)*** *Histopathological analysis of Day12 collected skin tissues from MPXV-infected mice topically treated with IRAK4-IN-6, SM-7368, and KRAS inhibitor-10. Representative H&E-stained images were captured using a 10X objective lens. MPXV-infected (vehicle-treated) mouse skin showed scab formation, leading to skin erosion (indicated by black arrows), with heavy inflammatory cell infiltrate. Drug treatment resulted in the resolution of these Mpox skin lesions with reduced inflammation. Labels "E" and "D" indicate the epidermis and dermis layers, respectively, as general reference points for skin morphology.* ***(C)*** *Immunohistochemistry staining of MPXV-infected mouse skin sections harvested on Day 12 post-infection. Tissue sections were stained for MPXV (MPXV A4L) (red) and DAPI (blue).* ***(D)*** *Viral antigen-positive cells were quantified, and percent infectivity was calculated for the graph. Statistical analysis was performed using ANOVA, followed by Tukey's post hoc test (*, $P < 0.05$; **, $P < 0.01$; ***, $P < 0.001$; ****, $P < 0.0001$).*

SUPPLEMENTARY TABLES

Supplementary Table 1. This table includes all 138 drug compounds identified in the primary screening, along with their target pathways and molecular structures.

Supplementary Table 2.

REAGENT/RESOURCE	SOURCE	IDENTIFIER
**Antibodies**		
Mpox virus A4L antibody [HL2555]	GeneTex	Cat#GTX638927
Phospho-NF-κB p65 (Ser536) (93H1) Rabbit mAb	Cell Signaling	Cat#3033
NF-κB p65 (D14E12) XP® Rabbit mAb	Cell Signaling	Cat#8242
Phospho-STING (Ser366) (E9A9K) Rabbit mAb	Cell Signaling	Cat#50907
STING (D2P2F) Rabbit mAb	Cell Signaling	Cat#13647
EpCAM (D9S3P) Rabbit mAb #14452	Cell Signaling	Cat#14452
Cleaved Caspase-3 (Asp175) Antibody #9661	Cell Signaling	Cat#9661
Goat anti-Mouse IgG (H+L) Cross-Adsorbed Secondary Antibody, Alexa Fluor 555	Thermo Fisher Scientific	Cat#A-21422
Goat anti-Rabbit IgG (H+L) Cross-Adsorbed Secondary Antibody, Alexa Fluor™ 488	Thermo Fisher Scientific	Cat#A-11008
Monoclonal Anti-Beta-Actin, Clone AC-74 produced in mouse	MilliporeSigma	Cat#A2228
**Bacterial and Virus Strains**		
hMPXV/USA/MA001/2022 (Lineage B.1, Clade IIb)	BEI Resources	Cat#NR-58622
**Chemicals, Peptides, and Recombinant Proteins**		
Regular Fetal Bovine Serum	Corning	Cat#35010CV
Eagle's Minimum Essential Medium (MEM)	Corning	Cat#10009CV
Penicillin-Streptomycin (10,000 U/mL)	Gibco	Cat#15140122
L-Glutamine (200 mM)	Gibco	Cat#25030081
MEM Non-Essential Amino Acids Solution(100X)	Gibco	Cat#11140050
KGM™ Gold Keratinocyte Growth Medium BulletKit™	Lonza	Cat#00192060
DMEM/F-12, HEPES	Thermo Fisher Scientific	Cat#11330032
SM-7368	MedChemExpress	Cat#HY-116626
IRAK4-IN-6	MedChemExpress	Cat#HY-130253
KRAS inhibitor-10	MedChemExpress	Cat#HY-138295
Tecovirimat	Selleckchem	CAT#S3380
Methanol, Optima™ LC/MS Grade, Fisher Chemical	Fisher Scientific	Cat#A456-500
Dimethyl sulfoxide	MilliporeSigma	Cat#D2650
16% Paraformaldehyde (formaldehyde) aqueous solution	Electron Microscopy Sciences	Cat#15710
Dulbecco's Phosphate-Buffered Salt Solution 1X	Corning	Cat#21030CV
DAPI (4',6-Diamidino-2-Phenylindole, Dihydrochloride)	Thermo Fisher Scientific	Cat#D1306
Corning™ Cell Culture Phosphate Buffered Saline (1X)	Thermo Fisher Scientific	Cat#MT21040CV

Bovine Serum Albumin	MilliporeSigma	Cat#A9418
Normal Donkey Serum	Jackson ImmunoResearch	Cat#017-000-121
Normal Goat Serum	Cell Signaling	Cat#5425S
Triton-X 100	MilliporeSigma	Cat#T9284
**Commercial Assays**		
CellTiter-Glo Luminescent Cell Viability Assay	Promega	Cat#G7570
**Experimental Models: Cell Lines**		
VERO C1008 [Vero 76, clone E6, Vero E6]	ATCC	Cat#CRL-158
ARPE-19	ATCC	Cat#CRL-2302
VK2/E6E7	ATCC	Cat#CRL-2616
NHEK-Neo – Human Epidermal Keratinocytes, Neonatal, Pooled	Lonza	Cat#00192906
**Software and Algorithms**		
GraphPad Prism 10	GraphPad	N/A
Multi-Point Tool (Cell Counter)	ImageJ	N/A
BioRender	BioRender	N/A

**COMPOUND DETAILS**	**MOLECULAR FORMULA/SEQUENCE**	**CAS#**	**STRUCTURE**
SM-7368	$C_{25}H_{22}O_{10}$	380623-76-7	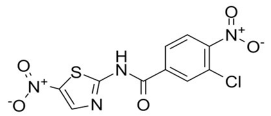
IRAK4-IN-6	$C_{25}H_{32}N_{10}O_2$	2454244-02-9	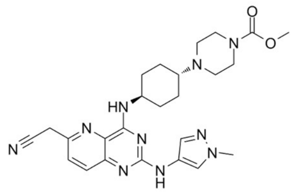
KRAS inhibitor-10	$C_{30}H_{37}N_3O_5$	2578876-75-0	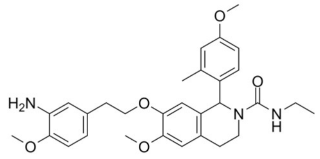

Niclosamide	$C_{13}H_8Cl_2N_2O_4$	50-65-7	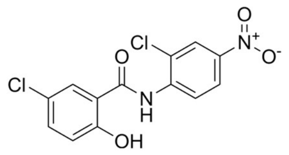
Tecovirimat	$C_{19}H_{15}F_3N_2O_3$	869572-92-9	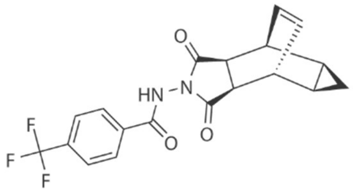
